# Torsadogenic Risk of Antipsychotics: Combining Adverse Event Reports with Drug Utilization Data across Europe

**DOI:** 10.1371/journal.pone.0081208

**Published:** 2013-11-20

**Authors:** Emanuel Raschi, Elisabetta Poluzzi, Brian Godman, Ariola Koci, Ugo Moretti, Marija Kalaba, Marion Bennie, Corrado Barbui, Bjorn Wettermark, Miriam Sturkenboom, Fabrizio De Ponti

**Affiliations:** 1 Department of Medical and Surgical Sciences, Alma Mater Studiorum - University of Bologna, Bologna, Italy; 2 Division of Clinical Pharmacology, Karolinska Institute, Stockholm, Sweden; 3 Strathclyde Institute of Pharmacy and Biomedical Sciences, Strathclyde University, Glasgow, United Kingdom; 4 Clinical Pharmacology Unit, University of Verona, Verona, Italy; 5 Republic Institute for Health Insurance, Belgrade, Serbia; 6 Information Services Division, NHS National Services Scotland, Edinburgh, United Kingdom; 7 WHO Collaborating Centre for Research and Training in Mental Health and Service Evaluation, Department of Public Health and Community Medicine, Section of Psychiatry, University of Verona, Verona, Italy; 8 Centre for Pharmacoepidemiology, Karolinska University Hospital, Solna, Stockholm, Sweden; 9 Erasmus University Medical Centre, Rotterdam, The Netherlands; Universidad de Valladolid, Spain

## Abstract

**Background:**

Antipsychotics (APs) have been associated with risk of torsade de Pointes (TdP). This has important public health implications. Therefore, (a) we exploited the public FDA Adverse Event Reporting System (FAERS) to characterize their torsadogenic profile; (b) we collected drug utilization data from 12 European Countries to assess the population exposure over the 2005-2010 period.

**Methods:**

FAERS data (2004-2010) were analyzed based on the following criteria: (1) ≥4 cases of TdP/QT abnormalities; (2) Significant Reporting Odds Ratio, ROR [Lower Limit of the 95% confidence interval>1], for TdP/QT abnormalities, adjusted and stratified (Arizona CERT drugs as effect modifiers); (3) ≥4 cases of ventricular arrhythmia/sudden cardiac death (VA/SCD); (4) Significant ROR for VA/SCD; (5) Significant ROR, combined by aggregating TdP/QT abnormalities with VA and SCD. Torsadogenic signals were characterized in terms of signal strength: from Group A (very strong torsadogenic signal: all criteria fulfilled) to group E (unclear/uncertain signal: only 2/5 criteria). Consumption data were retrieved from 12 European Countries and expressed as defined daily doses per 1,000 inhabitants per day (DID).

**Results:**

Thirty-five antipsychotics met at least one criterium: 9 agents were classified in Group A (amisulpride, chlorpromazine, clozapine, cyamemazine, haloperidol, olanzapine, quetiapine, risperidone, ziprasidone). In 2010, the overall exposure to antipsychotics varied from 5.94 DID (Estonia) to 13.99 (France, 2009). Considerable increment of Group A agents was found in several Countries (+3.47 in France): the exposure to olanzapine increased across all Countries (+1.84 in France) and peaked 2.96 in Norway; cyamemazine was typically used only in France (2.81 in 2009). Among Group B drugs, levomepromazine peaked 3.78 (Serbia); fluphenazine 1.61 (Slovenia).

**Conclusions:**

This parallel approach through spontaneous reporting and drug utilization analyses highlighted drug- and Country-specific scenarios requiring potential regulatory consideration: levomepromazine (Serbia), fluphenazine (Slovenia), olanzapine (across Europe), cyamemazine (France). This synergy should be encouraged to support future pharmacovigilance activities.

## Introduction

Antipsychotics (APs) represent a heterogeneous pharmacological class in terms of molecular targets [1], although several studies have challenged whether this multifaceted profile actually translates into clinical differences (i.e., effectiveness) between first- and second- generation agents (FGAs and SGAs), in the past referred to as “typical” and “atypical” drugs, respectively [2-4]. This uncertainty poses emerging clinical implications for psychiatrists when considering the appropriate therapy, which should be tailored to the individual [5-7].

The interest on this therapeutic class has strongly increased in the last two decades for several reasons, especially when new SGAs became available and were presented as having better benefit-risk profiles as compared to the older FGAs, namely a reduced risk of extra-pyramidal symptoms. Therefore, increased consumption was recorded throughout all age groups, ethnic groups and therapeutic indications, including off-label use [8,9]. The rapid changes in the uptake of these drugs require adequate monitoring of actual adverse effects in the real population to support future decision making. Thus, spontaneous reporting of adverse events represents a ready-to-use source of data to be analyzed for an early and timely identification of risks [10].

From a safety standpoint, the key topic has shifted from extra-pyramidal side effects to the novel aspect of the “cardio-metabolic” risk and cardiovascular toxicity, for which great interest has recently emerged [11]. Whilst there is consensus in assessing the metabolic risk and related cardiovascular complications (e.g., olanzapine is more strongly associated with weight gain as compared to other SGAs [12]), QT prolongation and subsequent arrhythmia-related events (i.e., Torsade de Pointes, TdP) represent the most important cardiac aspect of antipsychotics [13,14]. In the past, SGAs were perceived as having a favorable arrhythmogenic profile; however, this benefit has been recently challenged by different pharmacovigilance analyses on spontaneous reporting systems [15-17], which have also been confirmed by subsequent pharmacoepidemiological studies [18,19]. As a consequence, public lists of drugs with TdP liability collated by the Arizona CERT (http://crediblemeds.org/, a reference standard for clinicians) are constantly being updated and now include a number of antipsychotics.

Data on antipsychotic use can provide an additional perspective to regulators and clinicians in assessing the possible consequences of side effects of drugs by mapping the level of risk associated with antipsychotic exposure. Although several drug utilization studies have been conducted with different aims, especially in the US to assess the impact of regulatory warnings on the prescription pattern of antipsychotics [20-23], to the best of our knowledge, no detailed cross-national comparisons have been recently carried out in Europe. 

On these grounds, the principal aims of this study were twofold: 1) to characterize torsadogenic signals of antipsychotics emerging from the largest publicly available pharmacovigilance database, the FDA Adverse Event Reporting System (FAERS); 2) to offer the public health perspective of these pharmacovigilance signals (i.e., the population exposure) by collecting drug utilization data from 12 European Countries. Subsequently, notify the authorities in pertinent countries where there are potential concerns.

## Methods

### Ethics Statement

All data analyses performed in this retrospective study are based on anonymized data, which did not allow the precise identification of individual patients. The study dealt with two independent data sources, namely (a) spontaneous reports, which are publicly available from the FAERS database and (b) drug utilization data, which can be accessed by other researchers only upon request to relevant personnel. Therefore, submission to and approval by the Institutional Review Board was not required.

### Pharmacovigilance data source

By virtue of its large population coverage (including all US reports and rare/serious events from European Countries) and free availability since 2004, FAERS is an attractive source to detect emerging safety signals and a valuable tool to explore the worldwide reporting pattern of rare Adverse Drug Reactions (ADRs) such as TdP [15]. As a matter of fact, approximately 800,000 reports are entered into FAERS each year, of which almost a half is submitted by non-US Countries (e.g., European areas) and consumers. In addition, interest is emerging in using FAERS to obtain reliable within-class comparisons [24].

Before performing the analysis, a number of issues have been considered to obtain a reliable dataset: drug and event codification through *ad hoc* and standardized (MedDRA) archives, respectively; duplicate detection; management of missing data [25]. As previously detailed [15,26], the 2004-2010 period was analyzed through a record-linkage strategy of different files: DEMO (demographic data), DRUG (the analysis was restricted to APs reported as suspect or interacting), REACTION and OUTCOME (to assess the seriousness of the event; i.e., whether or not the ADR resulted in death or life-threatening conditions).

### Organizing pharmacovigilance data

In a recent study, we described the consensus process to define the torsadogenic potential of drugs and extract relevant cases of interest [27]. In summary, a wide range of clinical events with decreasing drug-attributable risk for TdP was collected: 1) TdP, 2) QT interval abnormalities (including QT prolongation), 3) Ventricular Arrhythmia (VA, including ventricular tachycardia and fibrillation) and 4) Sudden Cardiac Death (SCD). Building on this previous approach, in the present study, these clinical events were combined as follows: (a) TdP reports were analyzed with QT abnormalities as they are strongly intertwined with a high degree of drug-attributable risk (TdP is a so-called designated medical event, i.e., a rare and serious event with high drug-attributable component [28]); (b) VA reports were separately analyzed with SCD events as they are not strictly correlated with TdP and carry a reduced degree of drug-attributable risk [29].

Based on these events and considering the clinical scenario in which TdP usually occurs (i.e., the so called “reduced repolarization reserve” due to multiple risk factors such as concomitant drugs with TdP liability [30]), the following criteria/parameters (with relevant thresholds) were defined to analyze pharmacovigilance data:

1 Number of cases of TdP/QT abnormalities (at least 4 cases);
2 Significant Reporting Odds Ratio, ROR (i.e., Lower Limit of the 95%CI>1 and at least 4 cases) for TdP/QT abnormalities, adjusted for confounders (e.g., concomitant Class I/III antiarrhythmic drugs) in the stratum without drugs listed by Arizona CERT (effect modifiers) [27];
3 Number of cases of VA/SCD (at least 4 cases);
4 Significant ROR for VA/SCD, unadjusted and un-stratified;
5 Significant unadjusted and un-stratified ROR that persists throughout a cumulative approach (i.e., from TdP to SCD).

In order to analyze drug utilization data in a public health perspective, the following groups were created to characterized signals in terms of strength, based on the consistency of the aforementioned criteria:

A Antipsychotics with very strong torsadogenic signal (i.e., consistency among all criteria): all criteria exceeded relevant thresholds;
B Antipsychotics with strong torsadogenic signal (i.e., the majority of criteria are fulfilled): at least 4 out of 5 criteria exceeded relevant thresholds;
C Antipsychotics with weak/moderate torsadogenic signal (i.e., criteria not homogeneously fulfilled): at least 3 out of 5 criteria exceeded relevant thresholds;
D Antipsychotics with unclear/uncertain torsadogenic signal (i.e., the majority of criteria are not fulfilled): only 2 (or less) out of 5 criteria exceeded relevant thresholds.

### Drug utilization data collection

After safety signals are identified from FAERS, drug utilization data allow us to map the risk derived from exposure to antipsychotic drugs in each Country. Collection of drug utilization data on antipsychotics requires careful consideration of the available sources across Europe, including characteristics of databases (e.g., data quality, population coverage, data access), limitations and aims of the research [31]. In this study, drug utilization data were collected from administrative databases through health authorities and health insurance personnel across Europe. Consistent and reliable data were obtained from twelve European Countries, which allowed adequate estimation of European population exposure and represented differences in geography and financing of healthcare: they comprised Northern (Sweden, Norway and Scotland), Southern-Western (Spain [Catalonia], Austria, France, Italy) as well as Central-Eastern (Croatia, Serbia, Slovenia, Estonia, Lithuania) European Countries and regions. In addition, differences in the method of financing healthcare: taxation (Italy, Norway, Scotland, Spain [Catalonia], Sweden) and health insurance based (Austria, Croatia, Estonia, France, Lithuania, Serbia and Slovenia). 

Total dispended data (ATC code: N05A, excluding Lithium) were expressed as defined daily doses (DDD) per 1,000 inhabitants per day (DDD/TID, now referred to as DID) for the 2005-2010 period (with some exceptions detailed in the relevant table). Despite limitations, the DDD system is a recognized tool for standardizing antipsychotic doses in drug utilization research [32], and is currently recommended by the WHO for international drug utilization studies [33]. In the analyses, we considered a detectable use of at least 0.01 DID. The mean DID value was used to estimate the actual population exposure over the period of interest, in line also with pharmacovigilance data. A time trend analysis was also carried out, where appropriate. Data were re-validated with data providers to enhance their accuracy. A structured inventory of drug consumption databases available across Europe (with relevant details on data access and characteristics) has been recently provided within the PROTECT (Pharmacoepidemiological Research on Outcomes of Therapeutics by a European Consortium) project (http://www.imi-protect.eu/frameworkRep.shtml). Moreover, Table **S1** provided details of the databases used in our study. With only a few exceptions, administrative databases regards reimbursed prescriptions in ambulatory care and covered the entire population.

## Results

### Pharmacovigilance analyses

Over the 7-year-period of the analysis, 37 antipsychotics received at least one report in FAERS for the events of interest; 35 of them fulfilled at least one criterium for signal characterization. Table **1** provides a synopsis of pharmacovigilance criteria and their combination for signal characterization. Group A (i.e., antipsychotics that fulfilled all criteria) included 9 agents, 6 of which are SGAs. Of these, only cyamemazine is not included in any Arizona CERT lists (as of January 4^th^, 2013). Two agents were already included in List I (i.e., chlorpromazine and haloperidol), 5 SGAs in List II, amisulpride as the only compound in List III. Group B included 7 drugs (all FGAs, except droperidol), with only droperidol listed by Arizona CERT in List I. Group C comprised 13 agents, with paliperidone and pimozide recorded in Arizona CERT Lists. Eight antipsychotics were categorized in Group D.

**Table 1:** Synopsis of results from FAERS (2004-2010) according to criteria combined for signal characterization.

**Antipsychotic**	**Criterium 1**	**Criterium 2**	**Criterium 3**	**Criterium 4**	**Criterium 5**	**AZCERT List #**	**Signal characterization**
cyamemazine	x	x	x	x	x		A
olanzapine	x	x	x	x	x	II	A
amisulpride	x	x	x	x	x	III	A
chlorpromazine	x	x	x	x	x	I	A
clozapine	x	x	x	x	x	II	A
haloperidol	x	x	x	x	x	I	A
quetiapine	x	x	x	x	x	II	A
risperidone	x	x	x	x	x	II	A
ziprasidone	x	x	x	x	x	II	A
bromperidol	x		x	x	x		B
chlorprothixene	x		x	x	x		B
droperidol	x		x	x	x	I	B
fluphenazine	x		x	x	x		B
levomepromazine	x		x	x	x		B
prothipendyl	x		x	x	x		B
zuclopenthixol	x		x	x	x		B
paliperidone	x	x	x			II	C
aripiprazole	x	x	x				C
pimozide	x		x		x	I	C
flupentixol			x	x	x		C
loxapine			x	x	x		C
melperone			x	x	x		C
perazine			x	x	x		C
periciazine			x	x	x		C
perphenazine			x	x	x		C
pipamperone			x	x	x		C
prochlorperazine			x	x	x		C
sulpiride			x	x	x		C
zotepine			x	x	x		C
promazine			x		x		D
acepromazine			x				D
asenapine			x				D
tiapride			x				D
trifluoperazine			x				D
sultopride					x		D
levosulpiride							D
pipotiazine							D

Within each criterium, drugs are listed by considering the number of criteria fulfilled from 1 to 5 and the alphabetical order.

# based on the website http://crediblemeds.org/ (as of January 4^th^, 2013).

Only antipsychotics with at least one report for the event of interest are shown.

“X” indicates that the drug fulfilled relevant criterium.

Quetiapine (18,070), olanzapine (11,622) and clozapine (10,082) were the most frequently reported overall and also received the highest number of reports for the events of interest, namely group TdP/QT and VA/SCD (Table **2**). Notably, for most antipsychotics, also European reports have been submitted to FAERS and, in most of the cases, European Countries for which drug utilization were available have largely contributed (e.g., France). Concerning Group A drugs, cyamemazine (79.8%) and amisulpride (73.5%) received the highest proportion of reports from Europe. By contrast, quetiapine (75.7%) and risperidone (71.3%) mainly received US reports. As regards Group B agents, with the exception of fluphenazine, reports were mostly submitted by European Countries. Paliperidone and aripiprazole (Group C) received the majority of reports from US, whereas Group D agents from Europe (especially pipamperone and melperone). France, Germany and UK were the most frequent European reporter Countries.

**Table 2:** Overview of FAERS data with key information on reporter Country.

**Antipsychotic**	**TdP/QT cases**	**VA/SCD cases**	**Total reports $**	**US reports # (% of tot. Reports)**	EU* reports # (% of tot. Reports)	**Top 5 European Reporter Countries (%)**
cyamemazine	11	40	362	0.6	79.8	FRA (96.5); DNK (1.4); GER (1.0); UK (0.7); ITA (0.4)
olanzapine	189	712	11,622	43.8	26.6	FRA (23.3); UK (20.00); GER (18.3); SWI (7.4); SPA (4.2)
amisulpride	25	36	302	1.0	73.5	UK (31.5); FRA (28.4); GER (17.1); SWI (8.1); ITA (5.9)
chlorpromazine	14	69	611	28.3	20.3	FRA (47.6); UK (27.4); IRL (8.1); ITA (4.8); GRE (2.4)
clozapine	178	900	10,082	36.0	29.4	UK (58.1); GER (9.6); IRL (9.0); FRA (8.4); SWI (2.6)
haloperidol	125	239	3,582	20.6	50.9	FRA (30.2); ITA (20.7); GER (18.4); UK (8.7); SPA (6.9)
quetiapine	186	934	18,070	75.7	11.3	GER (25.4); UK (21.8); SWI (12.8); DNK (5.5); ITA (4.6)
risperidone	151	486	8,381	28.3	38.1	UK (25.1); FRA (25.0); GER (19.5); ITA (6.6); SPA (5.2)
ziprasidone	167	161	2,775	71.3	5.3	GER (37.0); SPA (11.6); ITA (8.2); DNK (6.2); GRE (4.8)
bromperidol	7	8	25		36.0	GER (55.6); BEL (33.3); FRA (11.1)
chlorprothixene	4	23	100	1.0	70.0	GER (51.4); DNK (21.4); SWI (7.1); NOR (4.3); SWE (2.9)
droperidol	10	20	131	26.0	37.4	FRA (85.7); UK (4.1); SWI (2.0); NOR (2.0); NLD (2.0)
fluphenazine	8	22	258	45.3	22.9	FRA (37.3); UK (25.4); CRO (20.3); SPA (6.8); ITA (5.1)
levomepromazine	6	39	272		61.8	FRA (47.6); SWI (13.1); UK (7.1); AUT (7.1); ITA (4.8)
prothipendyl	6	13	94		87.2	GER (75.6); AUT (12.2); DNK (7.3); ITA (2.4); FRA (2.4)
zuclopenthixol	6	16	112	3.6	73.2	FRA (31.7); UK (15.9); SWE (9.8); SWI (7.3); ITA (6.1)
paliperidone	11	46	1,702	77.2	13.4	GER (41.7); SPA (12.3); ITA (11.4); SWI (6.1); GRE (4.8)
aripiprazole	46	230	7,258	58.4	17.3	UK (28.2); FRA (24.8); GER (18.8); ITA (3.6); SWI (3.4)
pimozide	16	6	77	15.6	39.0	FRA (40.0); GER (23.3); UK (10.0); ITA (10.0); SPA (3.3)
flupentixol	2	9	84	2.4	76.2	SWI (21.9); GER (21.9); UK (18.8); FRA (10.9); DNK (9.4)
loxapine	3	22	171	11.7	70.8	FRA (97.5); DNK (2.5)
melperone	─	5	37		81.1	GER (86.7); FRA (6.7); UK (3.3); SWE (3.3)
perazine	─	8	52		59.6	GER (77.4); SWE (6.5); POL (6.5); FRA (3.2); DNK (3.2)
periciazine	─	7	41		61.0	FRA (72.0); UK (24.0); DNK (4.0)
perphenazine	─	13	134	47.8	24.6	UK (27.3); NOR (18.2); ITA (15.1); DNK (12.1); SPA (9.1)
pipamperone	1	15	145	2.8	88.3	GER (63.3); FRA (18.0); SWI (13.3); UK (2.3); DNK (2.3)
prochlorperazine	1	26	318	70.4	8.5	UK (77.8); IRL (11.1); SWI (3.7); FRA (3.7); DNK (3.7)
sulpiride	3	20	223	0.9	16.6	UK (32.4); FRA (24.3); GER (16.2); SPA (10.8); ITA (8.1)
zotepine	─	9	32	3.1	9.4	GER (66.7); PRT (33.3)
promazine	3	5	48	4.2	70.8	SWI (50.0); ITA (29.4); UK (8.8); FIN (5.9); CRO (5.9)
acepromazine	─	4	41	7.3	73.2	FRA (90.0); DNK (6.7); GER (3.3)
asenapine	2	7	623	100		
tiapride	1	7	95	3.2	75.8	FRA (79.2); ITA (5.6); GER (5.6); SWI (2.8); DNK (2.8)
trifluoperazine	─	7	104	48.1	12.5	UK (84.6); GER (7.7); FIN (7.7)
sultopride	1	3	13			
levosulpiride	1	─	8	12.5	62.5	ITA (100)
pipotiazine	─	1	7	14.3	85.7	FRA (83.3); UK (16.7)

$: excluding reports where antipsychotics were recorded as "concomitant". * including UK and Switzerland. # based on the information recorded in the field “Reporter Country”. The remaining fraction is related to missing data and other non -US/non-EU Countries (e.g., Japan). ─: no cases detected.

AUT: Austria; CRO: Croatia; DNK: Denmark; EST: Estonia; FIN: Finland; FRA: France; GER: Germany; GRE: Greece; IRL: Ireland; ITA: Italy; NLD: Netherlands; NOR: Norway; POL: Poland; PTR: Portugal; SCO: Scotland; SER: Serbia; SLO: Slovenia; SPA: Spain; SWI: Switzerland; UK: United Kingdom;

The full original data, including the results of the different disproportionality approaches (i.e., ROR values for each antipsychotic drug according to the event of interest), are provided as supplementary material (Table **S2**). These data have been used to characterize the strength of the pharmacovigilance signal.

### Drug Utilisation analyses

**Figure 1:**
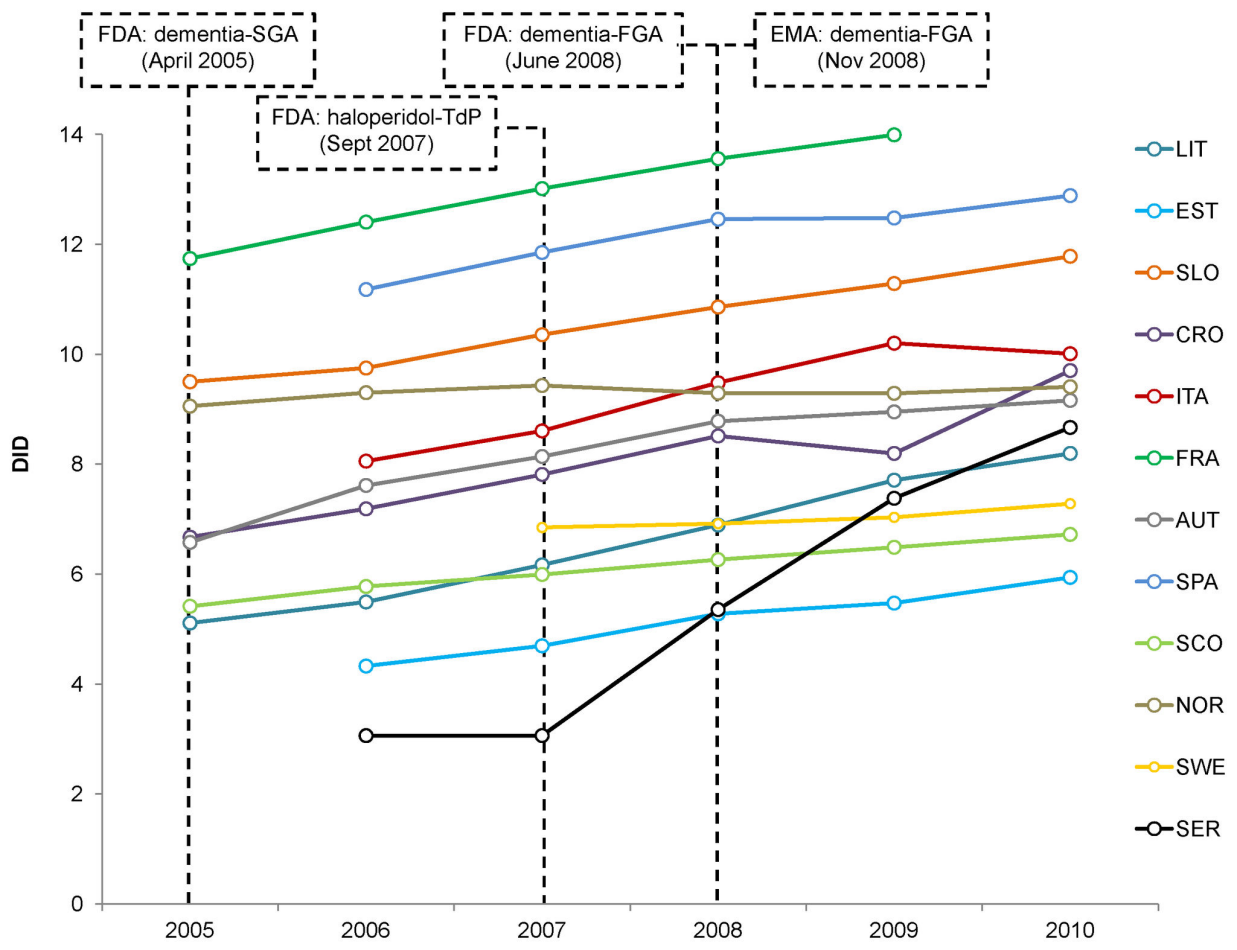
Trends in antipsychotic utilization according to data availability. AUT: Austria; CRO: Croatia; EST: Estonia; FRA: France; ITA: Italy; LIT: Lithuania; NOR: Norway; SPA: Spain (Catalonia); SCO: Scotland; SER: Serbia; SLO: Slovenia; SWE: Sweden; EMA: European Medicines Agency; FDA: Food and Drug Administration; FGA: first-generation antipsychotics; SGA: second-generation antipsychotics; TdP: Torsades de Pointes. Boxes indicate regulatory safety warnings on cardiovascular risk.

In 2010, the total exposure to antipsychotics (including, for instance, dixyrazine, thioridazine and sertindole, not included in or detected by the pharmacovigilance analysis) varied by a factor of approx 2, between Countries with the lowest (5.94 DID in Estonia) and highest (12.89 DID in Spain) use. In 2009, France peaked 13.99 DID (the latest available year of data collection). In all Countries, antipsychotic use increased over the period of interest, albeit with different trends among the different Countries. The increment was greatest in Central and Eastern European countries (+3.09 DID in Lithuania, +3.03 in Croatia) and especially in Serbia (+5.61 DID, +183%). This potentially reflects more drugs being placed on the reimbursement list, whereas in Northern European countries the consumption was more stable (+0.35 in Norway, +0.43 in Sweden) (Figure **1**). 

Table **3** provides an overview of drug utilization data on 37 antipsychotics arisen from the pharmacovigilance analyses and expressed in terms of mean DID over the study period. The mean exposure varied substantially: from France (12.92) and Slovenia (10.62) to Estonia and Lithuania (5.07 and 5.04, respectively). The mean use of SGAs was appreciably higher than the mean use of FGAs in all Countries, with the largest exposure to SGAs in Catalonia (Spain) (7.83). 

**Table 3:** Cross National Comparison: overview of drug utilization data on antipsychotics with at least one spontaneous report for the events of interest.

**Antipsychotics**	**LIT**	**EST**	**SLO**	**SER**	**CRO**	**ITA**	**FRA**	**AUT**	**SPA**	**SCO**	**NOR**	**SWE**
cyamemazine	─	─	─	─	─	─	2.74	─	─	─	─	─
olanzapine	1.25	0.73	2.34	0.04	1.60	1.96	1.71	1.53	2.53	2.05	2.89	1.97
amisulpride	0.38	0.06	0.26	─	0.01	0.20	0.76	0.33	0.38	0.41	0.12	─
chlorpromazine	─	0.17	─	0.37		0.15	0.06		0.03	0.59	0.08	0.01
clozapine	0.23	0.39	0.60	0.52	0.70	0.42	0.16	0.41	0.28	0.03	0.61	0.54
haloperidol	0.76	0.93	1.01	1.07	1.36	1.61	1.43	0.58	0.70	0.35	0.39	0.53
quetiapine	0.72	0.68	1.15	─	0.54	1.72	─	1.35	1.30	0.82	1.20	0.64
risperidone	0.65	0.46	1.59	1.02	0.85	0.91	2.46	1.38	2.41	0.76	0.85	1.05
ziprasidone	0.22	─	0.14	─	0.09	0.03	─	0.28	0.38	─	0.28	0.18
bromperidol	─	─	─	─	─	0.05	─	─	─	─	─	─
chlorprothixene	─	0.32	─	─	─	─	─	0.16	─	─	0.48	0.03
droperidol	─	─	─	─	─	─	─	─	─	─	─	─
fluphenazine	0.11	0.01	1.87	0.83	0.67	0.41	1.00		0.35	0.09	0.02	0.06
levomepromazine	─	0.09	0.15	2.58	0.19	0.08	0.16	0.12	0.12	0.02	0.34	0.20
prothipendyl	─	─	─	─	─	─	─	0.89	─	─	─	─
zuclopenthixol	0.11	0.33	0.34	0.03	0.06	0.14	0.37	0.11	0.17	0.31	0.50	0.61
paliperidone	─	─	0.08	─	─	─	─	─	─	─	─	0.03
aripiprazole	0.17	0.16	0.39	─	─	0.43	0.37	0.42	0.41	0.25	0.41	0.41
pimozide	─	─	─	─	─	0.07	0.09	0.02	0.05	0.02	─	0.01
flupentixol	0.06	0.19	0.13	─	─	─	0.28	0.03	─	0.09	0.28	0.22
loxapine	─	─	─	─	─	─	0.33	─	─	─	─	─
melperone	0.13	0.39	─	─	─	─	─	0.14	─	─	─	0.03
perazine	─	─	─	─	─	─	─	─	─	─	─	─
periciazine	─	─	─	─	─	0.05	0.15	─	0.05	0.02	─	─
perphenazine	─	0.06	─	─	─	0.04	0.01	─	0.11	0.01	0.66	0.45
pipamperone	─	─	─	─	─	─	0.11	─	─	─	─	─
prochlorperazine	─	─	─	─	─	─	─	─	─	─	0.12	0.01
sulpiride	─	0.05	0.23	─	0.35	0.02	0.12	0.05	0.14	0.19	─	─
zotepine	─	─	─	─	─	─	─	0.01	─	─	─	─
promazine	─	─	0.34	─	1.59	0.19	─	─	─	0.02	─	─
acepromazine	─	─	─	─	─	─	─	─	─	─	─	─
asenapine	─	─	─	─	─	─	─	─	─	─	─	─
tiapride	0.28	─	─	─	─	0.05	0.42	0.15	0.02	─	─	─
trifluoperazine	─	─	─	─	─	0.01	─	─	0.03	0.09	─	─
sultopride	─	─	─	─	─	─	─	─	─	─	─	─
levosulpiride	─	─	─	─	─	0.03	─	─	─	─	─	─
pipotiazine	─	─	─	─	─	─	0.18	─	0.03	─	─	─
**total**	**5.07**	**5.04**	**10.62**	**5.40**	**8.01**	**8.57**	**12.91**	**7.96**	**9.49**	**6.12**	**9.23**	**7.02**

─: no detectable use (i.e., DID<0.01, see methods).

Data are expressed in terms of mean DID for the 2005-2010 period, with the following exceptions: Estonia (2006-2010), Italy (2006-2010), France (2005-2009), Serbia (2006-2010), Spain (2006-2010) and Sweden (2007-2010).

**Figure 2:**
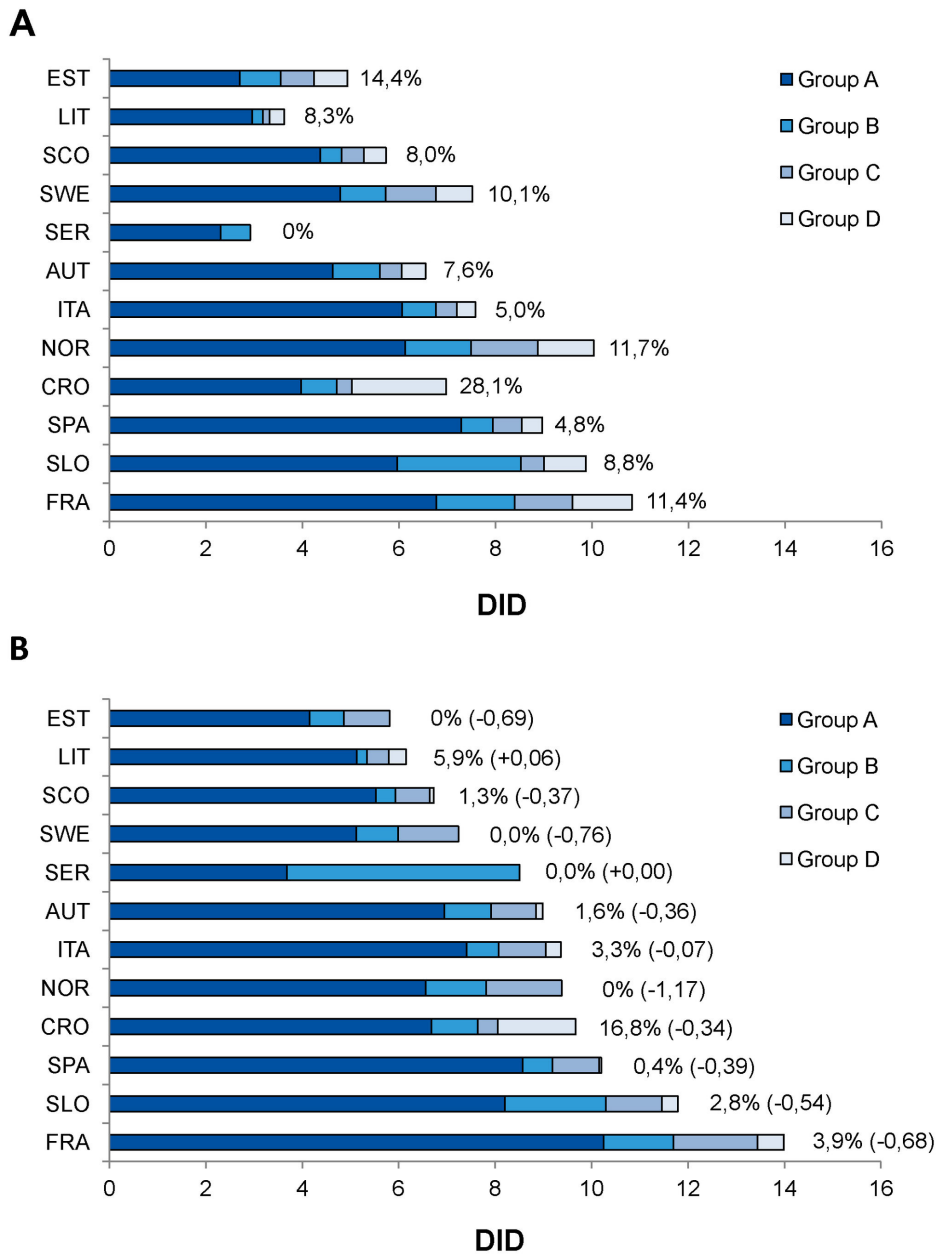
Antipsychotic utilization according to signal characterization: 2005 (Panel A) *versus* 2010 (Panel B). Countries were ranked by magnitude of 2010 antipsychotic consumption. Percentage: consumption of antipsychotics classified as group D/total antipsychotic consumption. In parenthesis changes in use of group D compounds (absolute values); + = increment in consumption; - = decrease in consumption.

Figure **2** provided the exposure to these 37 antipsychotics according to their characterization for torsadogenic signal. This time-trend analysis found that the overall utilization substantially increased in all Countries, especially in Serbia (+5.60) and France (+3.74). Only a limited increase in utilization was observed among Northern European Countries (+0.45 in Sweden, +0.51 in Norway). The consumption of SGAs increased in all Countries (+3.79 in France), although lower in Norway (+1.15) and Sweden (+0.67). By contrast, the use of FGAs showed a positive trend only in Serbia (+4.25). This positive trend in antipsychotic use was mainly driven by the increase in utilization of Group A agents: from +3.47 in France to +0.34 in Sweden, with substantial increment also in Croatia (+2.71), Austria (+2.32) and Slovenia (+2.24). In 2010 Spain (Catalonia) was the Country with the highest exposure to Group A agents (8.57); France ranked first in 2009 (10.25).

By contrast, the use of agents included in Group D was irregular and very low across Europe. In 2010, the magnitude of use was marked only in Croatia (1.62) and France (0.55). Generally, decreased utilization was recorded across all Countries, especially in Norway (-1.17) and Sweden (-0.76). An increase was found only in Lithuania (+0.06). 

There was generally limited use of antipsychotics included in Group B, as compared to Group A. However, the 2010 exposure to levomepromazine was high in Serbia (3.78). Appreciable use was also seen for fluphenazine, especially in Slovenia (1.63 in 2010), Serbia (1.02) and France (0.92 in 2009). The consumption of zuclopenthixol was considerably higher among Northern European Countries as compared to the remaining areas, with a peak of 0.31 and 0.60 (Norway in Sweden, respectively). Prothypendyl could be a specific concern in Austria (0.62) and chlorprothixene in Norway (0.48). 

The consumption of antipsychotics included in Group C was mainly driven by increasing utilization of aripiprazole, especially in Austria (0.75) and Italy (0.69) in 2010. Flupentixol (0.28 in Norway and France; 2010 and 2009, respectively) and perphenazine (0.59 and 0.44 in Norway and Sweden, in 2010) were appreciably used among Northern European Countries, melperone among in Central and Eastern European countries (peak of in 0.37 in Estonia in 2009). Sulpiride was used consistently across Europe, especially among Central and Eastern European Countries (0.41 in Croatia in 2010, where the consumption of promazine was also appreciable at 1.62).

**Figure 3:**
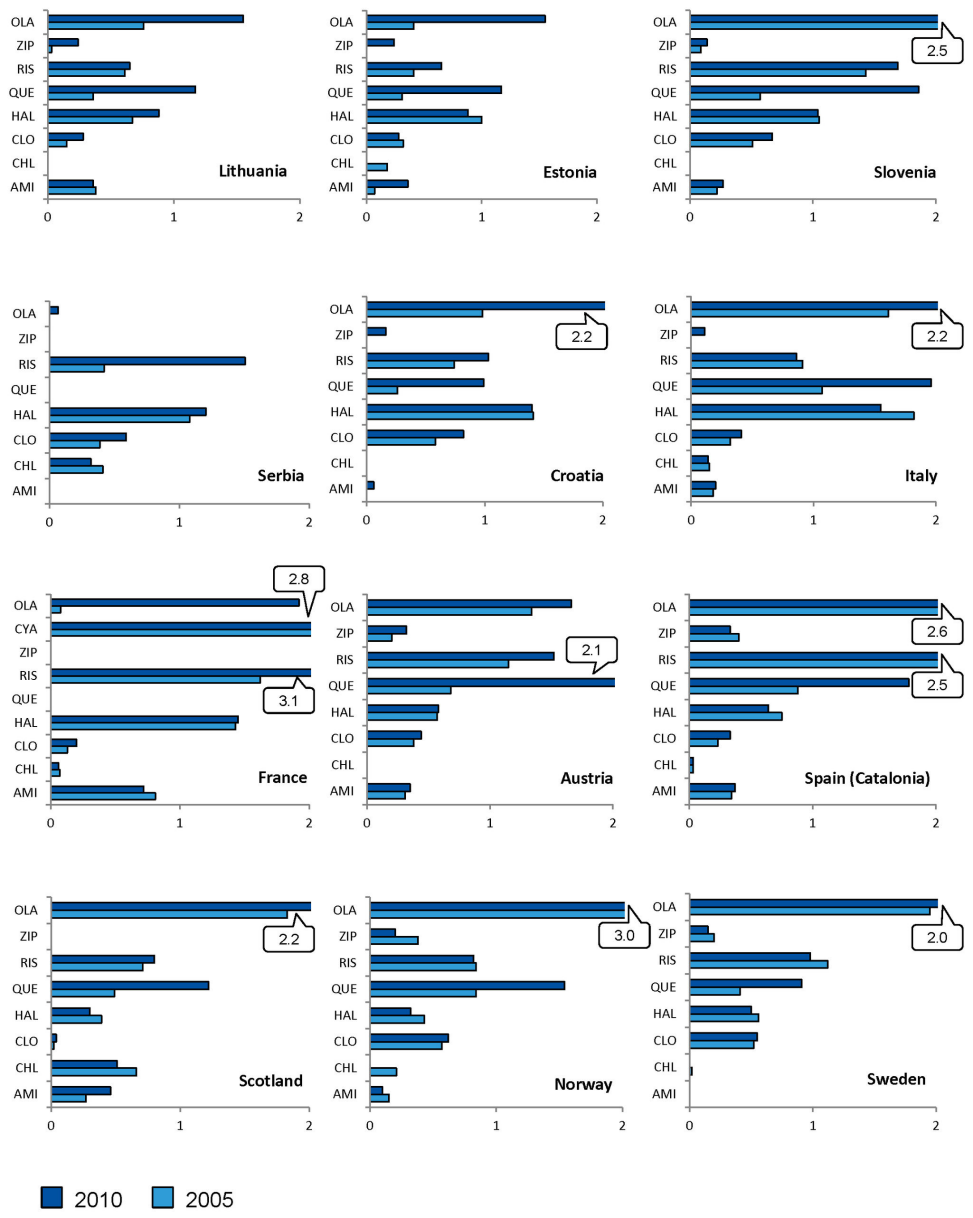
Country-by-Country Comparison in antipsychotic utilization included in Group A: 2005 *versus* 2010. Abscissa: DID. OLA: olanzapine; ZIP: ziprasidone; RIS: risperidone; QUE: quetiapine; HAL: haloperidol; CLO: clozapine; CHL: chlorpromazine; AMI: amisulpride; CYA: cyamemazine (shown only in France).

Figure **3** provides a drug-by-drug consumption of antipsychotics included in Group A. The use of these agents grew in all 12 Countries. In Serbia, the increment was caused by risperidone (+1.09); in France by olanzapine and risperidone (+1.84 and +1.47, respectively), whereas in 9 of the remaining 11 Countries, the trend was mostly driven by quetiapine (from +0.50 in Sweden to +1.40 in Austria). Olanzapine was consistently used across all Countries except in Serbia (0.07 in 2010), reaching 2.96 DID in Norway (2010) and showing increasing consumption in all Countries (+1.84 in France). Cyamemazine was selectively used only in France with large extent (2.81 in 2009). 

## Discussion

The 7-year-period analysis of FAERS characterized 35 antipsychotics with potential torsadogenic signal: 9 of them fulfilled all pharmacovigilance criteria to be labeled as very strong torsadogenic signals (i.e., Group A), with cyamemazine as the only agent currently not included in Arizona CERT lists (as of January 4^th^, 2013). Although the validity of the approach should be balanced against inherent limitations of spontaneous reporting system (see paragraph below), several issues may suggest its potential applicability in ranking torsadogenic signals for prioritization. For instance, the inclusion of antipsychotics already labeled in AZCERT lists in Group A such as haloperidol confirms the ability of our pharmacovigilance approach to accurately confirm actual safety signals. In addition, the case of cyamemazine underlines the sensitivity of the applied search strategy in revealing also Country-specific serious safety concerns from international archives such as FAERS; as a matter of fact, 96.5% of reports were submitted by France. Notably instead, quetiapine and risperidone received ¾ of reports from US despite being largely used in Europe. Demonstrating the validity of this pharmacovigilance approach to categorize torsadogenic signals is beyond the primary aim of this study and is correlated not only to the magnitude of drug use and its marketing penetration, but also to the efficiency of national pharmacovigilance systems, which may vary among Countries. Although, to the best of our knowledge, no study addressed this issue in FAERS, recent investigations in WHO-Vigibase and Eudravigilance found that 85% of reports were submitted by high-income Countries [34], with a commendable trend towards increased reporting activity of several national databases during recent years [35]. Because FAERS reports were mostly submitted by US and European high-income and upper middle-income Countries, we believe that FAERS data are highly informative of the global pattern of torsadogenic events. From a pharmacological standpoint, six out of ten in Group A are SGAs, which were originally perceived as safer in terms of torsadogenic potential [14]. Our findings, therefore, seem to challenge this conventional belief and are also in line with a recent multiple-treatment meta-analysis [36], thus making FAERS a reliable pharmacovigilance source to provisionally rank drugs for their TdP Liability. 

The Cross National Comparison on drug utilization highlighted an increasing exposure to antipsychotics across Europe, and found a peak of 13.99 DID in France in 2009, with appreciable differences in population exposure among the various European Countries. In 2010, the largest variation was recorded for Group A drugs: from 3.68 DID (Serbia) to 10.25 (France). These differences may at least partially be explained by local prescription habits, access to drugs e.g., hospital *vs* ambulatory, and differences in copayment levels especially among Central and Eastern European Countries [37,38]. In Serbia, the utilization of levomepromazine (3.78 DID in 2010) requires careful consideration, although it should be acknowledged that data from the Republic of Serbia included all reimbursed data (i.e., also hospital data). It is interesting to observe that our findings from Serbia are in line with past surveillance figures on outpatient consumption (2000-2004 period) [39], but differed from the most recent investigation (2005), which recorded 18.3 DID of antipsychotic consumption, including also hospital data [40]. The use of different data sources and providers could potentially explain these apparent inconsistencies. Fluphenazine is also largely used in Slovenia (1.63 in 2010). These findings may partially explain differences observed with a previous drug utilization study in Slovenia, which found lower antipsychotic consumption as compared to the Scandinavian Countries [41]. The case of olanzapine is a matter of concern for several Countries, especially in the light of increasing consumption throughout the period of interest. By contrast, in Serbia, only rarely is olanzapine dispensed to patients. When considering population exposure, fractional values should be also taken into account: for instance, although the overall consumption of antipsychotics is the highest in France, the use of Group D agents is also important (11.2% as compared to the overall use). Therefore, we believe that authorities in France should look more closely at prescription of Group A agents and consider policies such as educational interventions to reduce their utilization. Serbia should consider implementing measures to balance the utilization of Group B drugs (especially levomepromazine) towards potential therapeutic alternatives in Group D. Any strategy to reduce torsadogenic risk by APs should primarily consider that, especially in psychiatry, the overall risk-benefit profile of each agent is strongly influenced by specific patient response, both in terms of efficacy and safety. Consequently, health authorities should highlight all available information regarding the effectiveness and safety of different APs to help physicians make more informed decisions.

From regulatory and clinical standpoints, cross-national comparisons on drug utilization research allow the identification of specific scenarios requiring close post-marketing surveillance [42]. These studies should be encouraged by regulators and clinicians, especially for medicines intended for chronic use such as antipsychotics, because of the insufficient predictivity of premarketing investigations as compared to the real clinical setting [43]. Our study found some specific concerns that are exclusive for a given Country, which we believe require further research and discussion: this is the case for cyamemazine in France and prothipendyl in Austria. In particular, in the light of the high-quality pharmacovigilance system in France, we believe that *ad hoc* pharmacovigilance studies should be encouraged: detailed case-by-case assessment of reports and accurate calculation of reporting rates. Notably, different cross-sectional surveys [44-46] already explored the actual pattern of use (e.g., therapeutic indication and targeted population) and found that cyamemazine was one of the most frequently prescribed antipsychotic by virtue of its anxiolytic and sedative properties, in the context of mood disorders and other psychiatric disorders. The use of prothipendyl in Austria is in line with a study showing that it was the most frequently prescribed antipsychotic in nursing homes [47]. Further pharmacoepidemiological studies are needed to fully elucidate the prescribing pattern of prothipendyl.

From a research standpoint, drug utilization data have been shown to be a valuable data source for ecological studies [48]. Here, drug utilization data allow to approach pharmacovigilance data (derived by the clinical pharmacology perspective of single patients) in a population risk perspective. Specifically, we believe the role of drug utilization data is crucial in (1) measuring the public health impact of ADRs (mapping the pro-arrhythmic risk of a given drug), (2) interpreting pharmacovigilance data and (3) calculating the reporting rate (i.e., raw risk estimates after performing the causality assessment of cases obtained only from efficient pharmacovigilance systems) [49]. 

The large and steadily increasing use of antipsychotics in Europe, with considerable differences among Countries, implies different population exposure with different levels of risk. This joint analysis, although preliminary, demonstrates the synergy between drug utilization and adverse event databases to alert health policy personnel of potential future activities to reduce ADRs, especially for drugs associated with very strong torsadogenic signal. Both *ad hoc* initiatives (e.g., haloperidol [50,51]) or more general approaches for an entire pharmacological class could be hypothesized [52]. 

Drug utilization data also represents a key way forward to interpret pharmacovigilance data: the so-called “targeted pharmacovigilance approach” [27]. A list of antipsychotics that may tentatively be considered to carry a low torsadogenic risk should be based on: (a) “negative” pharmacovigilance data (i.e., none of the aforementioned criteria fulfilled); (b) long time on the market (at least 10 years); (c) detectable use (i.e., at least 0.01 DID) in at least 3 Countries. Based on our data, no antipsychotics met all these criteria and, therefore, they appear to carry an inherent torsadogenic risk. However, within Group D, the use of tiapride should not be overlooked: 0.39 in France (2009), 0.36 in Lithuania (2010), with 7 cases of VA/SCD recorded in FAERS (only criterium 4 was fulfilled) and 104 total reports. 

From an epidemiological standpoint, the large utilization of antipsychotics is a well known pharmaco-epidemiological burden, also for off-label indications [9]. Several studies on US and Canada prescriptions examined the impact of regulatory safety warnings and found only a slight decrease or even an increase of the antipsychotic use (especially SGAs in patients with dementia) [20-23]. Our data are also in line with a recent analysis on Australian data, which revealed a +217.7% increase in atypical antipsychotic dispensation over the 2000-2011 period, with olanzapine as the most frequently dispensed agent [53]. A previous European Cross National Comparison survey, performed on 12 Countries over the 2000-2005 period, highlighted large variability in antipsychotic exposure, but reported only a slight increase in their consumption [40].

Therefore, our study found that, notwithstanding recent evidence and regulatory safety warnings, the use of antipsychotics (and especially SGAs) continues to rise in Europe. Consequently, we believe there is need to implement effective initiatives of risk management to reduce antipsychotic medication prescriptions or promote appropriateness. 

### Limitations of the study

The clinical implications of this study should be carefully evaluated in the light of limitations affecting both pharmacovigilance and drug consumption data.

The analysis of spontaneous reports does not allow establish causality and is affected by the under-reporting; thus, no incidence can be calculated. In addition, there are several external factors that may influence the reporting [54-56]. In this respect, a number of safety warnings on the cardiovascular risk have been posted by the FDA that may have stimulated reporting: in 2005, SGAs received an alert on the risk of death in elderly patients with behavioral disturbances (http://www.fda.gov/Drugs/DrugSafety/PostmarketDrugSafetyInformationforPatientsandProviders/DrugSafetyInformationforHeathcareProfessionals/PublicHealthAdvisories/ucm053171.htm), extended in June 2008 (FDA) (http://www.fda.gov/Drugs/DrugSafety/PostmarketDrugSafetyInformationforPatientsandProviders/ucm107211.htm) and November 2008 (EMA) (http://www.ema.europa.eu/docs/en_GB/document_library/Report/2010/01/WC500054057.pdf) to FGAs; in 2007, haloperidol received a specific FDA alert on the risk of TdP/QT prolongation with relevant label changes (http://www.fda.gov/Drugs/DrugSafety/PostmarketDrugSafetyInformationforPatientsandProviders/DrugSafetyInformationforHeathcareProfessionals/ucm085203.htm). 

Although also serious and rare European reports are submitted to the FDA, our data may be affected by specific drug marketing penetration (e.g., drugs only marketed in Europe). In addition, the characterization of signals is dynamic so that a given antipsychotic may change groups depending on the availability of new pharmacovigilance data over time.

This study attempted to overcome the lack of information on exposed population in spontaneous reporting sources, by linking FAERS findings with drug consumption. These two data sources are independent and provide different perspectives, although they could be theoretically related and influenced by each other. As a matter of fact, it should be acknowledged that antipsychotics included in Group A are also largely and consistently used across Europe. 

Although pharmacovigilance and drug utilization data sources did not cover the same geographical area (only Europe for drug utilization and theoretically all Countries for pharmacovigilance), we used these two dataset for different purposes: (a) FAERS, the largest public pharmacovigilance database, to accurately classify and characterize torsadogenic signal by antipsychotics, and (b) European drug utilization data to map the overall antipsychotic use (i.e., estimate the European population exposure). 

Finally, with the exception of Scandinavian Countries [57] and now Scotland, record-linkage analyses with actual therapeutic indications, diagnoses and other clinical variables are challenging through reimbursement prescription databases. Nonetheless, we did not aim to assess whether differences arise among labeled and unlicensed indications or evaluate patients’ characteristics. 

## Conclusions

This targeted pharmacovigilance approach prioritized a group of 9 APs with a very strong torsadogenic signal and provide evidence that the European population is still largely exposed to them, especially SGAs. Notwithstanding the regulatory safety concerns, the use of SGAs has started to rise again among a number of European Countries during the last 6-year period. This potentially poses a public health concern in the acute management of arrhythmia-related events when antipsychotics are used outside the hospital setting.

This Cross National Comparison study highlighted (a) considerable differences in the level of population risk among the different European Countries in 2010 and (b) specific drug- or Country-related scenarios that should be considered by regulators and clinicians to establish tools for monitoring appropriateness: levomepromazine in Serbia, fluphenazine in Slovenia, cyamemazine in France, olanzapine across Europe.

We believe that a routine joint approach between spontaneous reporting and drug utilization analyses together with synergy among healthcare professionals, academia, regulators and industry should be encouraged for future pharmacovigilance activities such as post-authorization safety studies.

## Supporting Information

Table S1 **Details on administrative databases used to collect drug utilization data (with relevant references); original names of data sources, types of data and population coverage.**
(DOCX)

Table S2 **Original results from data mining of the FAERS database (2004-2010): number of cases and disproportionality analyses for all events of interest.** These data have been used to characterize the strength of the pharmacovigilance signal.
(DOCX)
