# Supplementary material for: Torsadogenic Risk of Antipsychotics: Combining Adverse Event Reports with Drug Utilization Data across Europe
Source: PLoS One. 2013 Nov 20;8(11):e81208. doi: 10.1371/journal.pone.0081208 (PMC3835678; doi:10.1371/journal.pone.0081208)
Supplement: Table S1 — Details on administrative databases used to collect drug utilization data (with relevant references); original names of data sources, types of data and population coverage. (DOCX) [file pone.0081208.s001.docx]

**Table S1.** Details on administrative databases with relevant references.

| **Country** | **Characteristics** |
| --- | --- |
| Austria (1,2) | Internal data warehouse of the HVB (BIG), Cube HMSTAT, based on the "maschinelle Heilmittelabrechnung", which covers approximately 98% of the Austrian population. Only includes reimbursed prescriptions in ambulatory care |
| Croatia (3) | The Croatian Institute for Health Insurance (HZZO) database, owned by the Croatian Government, and the single provider of mandatory health care insurance for up to 98.4% of the inhabitants of Croatia. Only includes reimbursed prescriptions in ambulatory care |
| Estonia (4) | Estonian Health Insurance Fund. Only includes reimbursed prescriptions in ambulatory care (covers the entire population) |
| France (1,4) | Medic'am database (CNAM-TS database). Only includes reimbursed prescriptions in ambulatory care - Medic'am database (CNAM-TS database for salaried personnel covering 75% of the population) |
| Italy | Internal data warehouse from “flusso della tracciabilità”, a system recording all packages distributed by pharmacies (including OTC) and hospitals |
| Lithuania (4,5) | Electronic database of the Lithuanian Compulsory Health Insurance Information system. The database provides the complete reimbursed prescription medication history of patients, diagnosis (according IDC-10), physician prescriber as well as the pharmacy where the prescription was dispensed. Only includes reimbursed prescriptions in ambulatory care |
| Norway (4,6) | Norwegian Drug Wholesales Statistics (Wholesales Statistics), which resides in the Norwegian Institute of Public Health. Only includes reimbursed prescriptions in ambulatory care. Covers the entire population |
| Scotland (1,4,7) | National Health Services Scotland Warehouse (for reimbursed prescriptions). Only includes reimbursed prescriptions in ambulatory care. Covers the entire population |
| Serbia (8) | Republic of Serbia’s Health Insurance Fund database containing all reimbursed data covering the entire resident population in Serbia |
| Slovenia (4) | The National Institute of Public Health and Health Insurance Institute Prescription Database. Only includes reimbursed prescriptions in ambulatory care. Covers the entire insured population |
| Spain (Catalonia) (4,9) | DMART (Catalan Health Service) database (covers all patients in Catalonia). Only includes reimbursed prescriptions in ambulatory care |
| Sweden (10, 11) | National Swedish Pharmacy Register database containing data on reimbursed prescriptions, hospital dispensed data and OTC data. Covers the entire population |

**Supplementary References**

1. Godman B, Shrank W, Andersen M, et al. Comparing policies to enhance prescribing efficiency in Europe through increasing generic utilisation: changes seen and global implications. Expert Rev Pharmacoecon Outcomes Res 2010; 10: 707–22

2. Bucsics A, Godman B, Burkhardt T, et al. Influence of lifting prescribing restrictions for losartan on subsequent sartan utilisation patterns in Austria; implications for other countries. Expert Rev Pharmacoecon Outcomes Res 2012; 12: 809-19

3. Brkičic L, Vončina L, Sovic S, et al. Initiatives to improve prescribing efficiency for drugs to treat Parkinson’s Disease in Croatia; influence and future directions. Expert Rev Pharmacoecon Outcomes Res 2012; 12:373-84

4. Godman B, Shrank W, Andersen M, et al. Policies to enhance prescribing efficiency in Europe: findings and future implications. Frontiers Pharmacol 2011; 1 (141): 1-16 doi: 10.3389/fphar.2010.00141

5. Garuoliene K, Godman B, Gulbinovič J, et al. European countries with small populations can obtain low prices for drugs: Lithuania as a case history. Expert Rev Pharmacoecon Outcomes Res 2011; 11: 343-9

6. Godman B, Sakshaug S, Berg C, et al. Combination of prescribing restrictions and policies to engineer low prices to reduce reimbursement costs. Expert Rev Pharmacoecon Outcomes Res 2011; 11: 121–9

7. Bennie M, Godman B, Bishop I, et al. Multiple initiatives continue to enhance the prescribing efficiency for the proton pump inhibitors and statins in Scotland. Expert Rev Pharmacoecon Outcomes Res 2012; 12: 125-130

8 Kalaba M, Godman B, Vuksanovic A, et al. Possible ways to enhance renin-angiotensin prescribing efficiency: Republic of Serbia as a case history? Journal of Comparative Effectiveness Research 2012; 1:539-49

9. Coma A, Zara C, Godman B, et al. Policies to enhance the efficiency of prescribing in the Spanish Catalan Region: impact and future direction. Expert Rev Pharmacoecon Outcomes Res 2009; 9: 569-81

10. Godman B, Wettermark B, Miranda J et al. Influence of multiple initiatives in Sweden to enhance ARB prescribing efficiency following generic losartan; findings and implications for other countries. International Journal of Clinical Practice 2013, Apr 8. doi: 10.1111/ijcp.12130. [Epub ahead of print]

11. Wettermark B, Hammar N, Fored M, et al. The new Swedish Prescribed Drug Register – Opportunities for pharmacoepidemiological research and experience from the first six months. Pharmacoepidemiol Drug Saf 2007;16:726-35
