# Supplementary material for: Torsadogenic Risk of Antipsychotics: Combining Adverse Event Reports with Drug Utilization Data across Europe
Source: PLoS One. 2013 Nov 20;8(11):e81208. doi: 10.1371/journal.pone.0081208 (PMC3835678; doi:10.1371/journal.pone.0081208)
Supplement: Table S2 — Original results from data mining of the FAERS database (2004-2010): number of cases and disproportionality analyses for all events of interest. These data have been used to characterize the strength of the pharmacovigilance signal. (DOCX) [file pone.0081208.s002.docx]

**Supplementary Table S2.** Data Mining of the FAERS database (2004-2010).

| **Antipsychotic** | **CRITERIUM 1: ≥4 cases TdP/QT (without AZCERT drugs#)** | **CRITERIUM 2: ROR (LL95%CI) for TdP/QT, adjusted stratified with ≥4 cases** | **CRITERIUM 3: ≥4 cases VA/SCD** | **CRITERIUM 4: ROR (LL95%CI) VA/SCD with ≥4 cases** | **CRITERIUM 5: ROR (LL95%CI) from TdP to SCD with ≥4 cases** |
| --- | --- | --- | --- | --- | --- |
| cyamemazine | 11 (6) | 15.48 (6.87-34.91) | 40 | 2.74 (1.92-3.82) | 3.24 (2.41-4.36) |
| olanzapine | 189 (121) | 7.74 (6.45-9.30) | 712 | 1.44 (1.34-1.56) | 1.67 (1.56-1.78) |
| amisulpride | 25 (10) | 43.94 (22.82-84.60) | 36 | 2.98 (2.05-4.25) | 5.00 (3.78-6.63) |
| chlorpromazine | 14 (8) | 12.93 (6.42-26.05) | 69 | 2.81 (2.15-3.62) | 3.11 (2.47-3.92) |
| clozapine | 178 (110) | 7.71 (6.35-9.35) | 900 | 2.18 (2.03-2.33) | 2.38 (2.23-2.54) |
| haloperidol | 125 (49) | 10.58 (7.95-4.10) | 239 | 1.58 (1.38-1.80) | 2.24 (2.01-2.50) |
| quetiapine | 186 (99) | 4.78 (3.91-5.85) | 934 | 1.21 (1.13-1.29) | 1.31 (1.23-1.39) |
| risperidone | 151 (79) | 6.96 (5.55-8.72) | 486 | 1.36 (1.24-1.49) | 1.63 (1.50-1.77) |
| ziprasidone | 167 (94) | 27.28 (22.06-33.72) | 161 | 1.36 (1.15-1.60) | 2.65 (2.36-2.98) |
| bromperidol | 7 (1) | n.a. | 8 | 10.38 (3.88-25.39) | 29.64 (13.32-65.98) |
| chlorprothixene | 4 (1) | n.a. | 23 | 6.59 (3.95-10.62) | 7.31 (4.70-11.37) |
| droperidol | 10 (3) | n.a. | 20 | 3.98 (2.34-6.35) | 5.87 (3.90-8.82) |
| fluphenazine | 8 (3) | n.a. | 22 | 2.06 (1.27-3.19) | 2.60 (1.78-3.80) |
| levomepromazine | 6 (1) | n.a. | 39 | 3.69 (2.56-5.20) | 3.92 (2.85-5.39) |
| pimozide | 16 (3) | n.a. | 6 | 1.86 (0.66-4.26) | 7.90 (4.82-12.96) |
| prothipendyl | 6 (3) | n.a. | 13 | 3.56 (1.81-6.40) | 5.01 (3.03-8.28) |
| zuclopenthixol | 6 (2) | n.a. | 16 | 3.68 (2.02-6.28) | 4.83 (3.03-7.70) |
| paliperidone | 11 (6) | 2.53 (1.13-5.65) | 46 | 0.61 (0.45-0.82) | 0.68 (0.53-0.89) |
| aripiprazole | 46 (19) | 1.78 (1.13-2.80) | 230 | 0.72 (0.63-0.82) | 0.78 (0.69-0.88) |
| flupentixol | 2 (1) | n.a. | 9 | 2.65 (1.17-5.30) | 2.98 (1.58-5.61) |
| loxapine | 3 (0) | n.a. | 22 | 3.26 (1.98-5.12) | 3.38 (2.21-5.17) |
| melperone | 0 | n.a. | 5 | 3.45 (1.05-8.91) | 3.09 (1.20-7.92) |
| perazine | 0 | n.a. | 8 | 4.01 (1.63-8.61) | 3.59 (1.69-7.63) |
| periciazine | 0 | n.a. | 7 | 4.54 (1.70-10.41) | 4.07 (1.80-9.18) |
| perphenazine | 0 | n.a. | 13 | 2.37 (1.23-4.21) | 2.12 (1.20-3.76) |
| pipamperone | 1 (0) | n.a. | 15 | 2.55 (1.39-4.36) | 2.45 (1.46-4.12) |
| prochlorperazine | 1 (1) | n.a. | 26 | 1.97 (1.26-2.94) | 1.83 (1.24-2.72) |
| sulpiride | 3 (2) | n.a. | 20 | 2.17 (1.30-3.45) | 2.27 (1.48-3.50) |
| zotepine | 0 | n.a. | 9 | 8.63 (3.52-19.37) | 7.73 (3.58-16.71) |
| acepromazine | 0 | n.a. | 4 | 2.39 (0.62-6.64) | 2.14 (0.76-5.99) |
| asenapine | 2 (2) | n.a. | 7 | 0.25 (0.10-0.52) | 0.29 (0.15-0.56) |
| levosulpiride | 1 (1) | n.a. | 0 | n.a. | n.a. |
| pipotiazine | 0 | n.a. | 1 | n.a. | n.a. |
| promazine | 3 (0) | n.a. | 5 | 2.57 (0.79-6.46) | 3.95 (1.85-8.44) |
| sultopride | 1 (0) | n.a. | 3 | n.a. | 8.78 (2.70-28.51) |
| tiapride | 1 (0) | n.a. | 7 | 1.76 (0.69-3.77) | 1.82 (0.88-3.75) |
| trifluoperazine | 0 | n.a. | 7 | 1.59 (0.62-3.41) | 1.43 (0.66-3.07) |

# this information was used to calculate the adjusted ROR in the stratum without AZCERT drugs (i.e., criterium 2).

TdP: Torsades de Pointes; QT: QT interval abnormalities; VA: ventricular arrhythmia; SCD: sudden cardiac death.

AZCERT: Arizona CERT website ([www.crediblemeds.org](http://www.crediblemeds.org)).

n.a.: not applicable due to the low number of cases (less than 4).
